# Supplementary material for: The sum of all parts: A multi-level exploration of racial and ethnic identity formation during emerging adulthood
Source: PLoS One. 2023 Apr 7;18(4):e0284275. doi: 10.1371/journal.pone.0284275 (PMC10081756; doi:10.1371/journal.pone.0284275)
Supplement: Attachment — Submitted filename: response to reviewers.docx [file pone.0284275.s001.docx]

Thank you for your helpful comments and suggestions for revision. We believe that the revisions have made this a stronger paper and a more clear contribution to the literature.

| Reviewer | Comment | Response |
| --- | --- | --- |
| Editor | 2. Please provide additional details regarding participant consent. In the ethics statement in the Methods and online submission information, please ensure that you have specified (1) whether consent was informed and (2) what type you obtained (for instance, written or verbal, and if verbal, how it was documented and witnessed). If your study included minors, state whether you obtained consent from parents or guardians. If the need for consent was waived by the ethics committee, please include this information. | Additional information about IRB approval and consent process has been added to the methods section. |
| Editor | 3. We noted in your submission details that a portion of your manuscript may have been presented or published elsewhere. [Figure 1 is the conceptual framework first created by Grilo et al. and used as the grounding framework for this study (2022)] Please clarify whether this [conference proceeding or publication] was peer-reviewed and formally published. If this work was previously peer-reviewed and published, in the cover letter please provide the reason that this work does not constitute dual publication and should be included in the current manuscript. | We have removed the figure from the publication and just discuss it and reference the previous publication in the manuscript. |
| Editor | 4. Please include a caption for figure 1. | Figure 1 has been removed the paper. |
| Editor | 5. Please include your tables as part of your main manuscript and remove the individual files. Please note that supplementary tables (should remain/ be uploaded) as separate "supporting information" files | The tables are now included as part of the main manuscript and not as separate files. |
| 1 | I think that this dataset represents a unique source of information, and the theoretical approach is a strong contribution to understanding multiracial identity development from multiple angles. My primary concerns are a lack of clarity around how health and life course factors fit into the interview approach and data. Although some of this may be fixed with revision or perhaps selecting quotes that are more representative of the data, it can also be indicative of the researchers imposing their own framework on data that doesn’t adequately reflect all parts of this framework (e.g., lack of health discussion in the results and yet is discussed as a major contribution of the paper). More methodological detail would assuage reader concerns regarding the latter. Below I outline my primary comments. I hope the authors find this feedback helpful. | Thank you for your comments about the contribution of this manuscript and thank you for your constructive feedback - we believe revisions have made this a stronger paper.   We have tried to clarify throughout the emphasis on life course factors and clarified the aims of the paper in the introduction and discussion. The results are also organized by different stages in the life course – childhood, and emerging adulthood. We also removed much of the conversation regarding health since that was not a focus on this particular manuscript. |
| 1 | 1. Although the conceptual framework by Grilo et al. is discussed in depth in a previous paper, and is based on the social ecological approach, it needs to be described further in the Introduction. What are the specific ways it expands on, or draws from, Bronfenbrenner’s approach? One question I had that is relevant to the current article: Why is identity based stress a causal influence on identification – is it not plausible that identification styles and how they are perceived can impact identity based stress (e.g., through identity invalidation)? What part(s) of this model is the current research drawing from or seeking to document? | Thank you. we added more explanation of the model into this paper and discussed why we added a life course component to the model. We also emphasized that this particular analysis and paper focuses on identity development and the many intersecting influences on that identity development throughout different stages of life. The components about health and stress are out of the scope of this particular analysis and we will focus on these sections in an additional manuscript. |
| 1 | 2. Operationalization and measurement of the life course aspect needs further clarification. | We added discussion on how we ask participants to reflect on different aspects of their life and how the identity process has changed over time. |
| 1 | 2a. Saying that the current study looks at influences on identification “throughout the life course” seems a bit overstated given such a restrictive age range (18-29). I recommend adjusting this language to be more reflective that the authors are studying emerging adults and adults. In the Results section it becomes obvious that participants were specifically instructed to retrospectively talk about their childhood. Additional detail with the interview prompts may help illuminate how the researchers are thinking about life course factors early on, or perhaps clarifying in the Introduction that the research will examine participants’ experiences from childhood to present. | We have clarified in the introduction and the methods sections that the research is among emerging adulthoods but that we ask them to reflect on experiences from childhood to present.  We also added the full interview guide as an appendix. |
| 1 | 2b. As another example of needing clarification on life course approaches, the Results section states, “These influential interactions were regarded as early, yet continuous, influences on their racial and ethnic identity” (pg. 17). Yet, none of the quotes in this section discuss identification in this way – most of the quotes are in the current tense. The summary on this section seems more tied to the themes in the “Life Course” section below it. | We have re-worked this section of the results section and moved much of this to the next section on life course. We also changed how we discussed these intersecting influences to be clear participants are reflecting on their childhood and then also the present as emerging adults. |
| 1 | 3. The authors discuss health and well-being at length in the abstract, introduction, and discussion, and even label it as a topic within the semi-structured interview. The word health never appears in the Results. Although I may have missed it, none of the quotes discuss health directly. There are references to feeling upset, or belonging, but most of the data center around identity development. | Thank you for bringing this to our discussion. We do have some data on health and wellbeing but because of the amount of data on identity development and intersecting influences on that development we decided to just focus on this piece of our data for this particular manuscript. We have taken out most of the references to health and wellbeing and that data will be included in a subsequent manuscript. |
| 1 | 4. There is insufficient detail regarding the semi-structured interview. Who conducted the interviews? What were the semi-structured prompts? In what ways were interviewers instructed to adapt or expand on certain topics? | These details about the interviews were added into the methods section. |
| 1 | - A few typos throughout the paper (e.g., page 1 “Understanding Using this framework”) | Thank you, the paper has been checked for typos. |
| 1 | - Multi-methods are usually used to refer to approaches that combine multiple methods – from my reading of the manuscript, qualitative analyses of interviews were used. Can the authors expand on how this is mult-method research or remove that claim from the abstract? | Thank you, the reference to mixed-methods has been removed from the abstract. |
| 1 | - The Discussion should include limitations of a top-down approach to the qualitative analysis (i.e., imposing a framework on the data). | This limitation was added with context to the discussion section. |
| 1 | To summarize, the themes were well described with supporting data. However, the paper’s Introduction, Discussion and Results are disjointed. For example, the Intro seems to emphasize identity development, structural factors, and influences on health. The Discussion emphasizes life course differences. Overall, the research would benefit from more clarification about the aims, how the described framework informed the current aims, and more detail regarding the methodological decisions. With revision and more transparency on the interviews and data analysis approach, the contribution could be improved. | Thank you for your help in creating a consistent story throughout the paper.  We have tried to emphasize the pieces on identity development and structural factors and removed the portions relating to the influences on health.   We hope that all of the revisions made have helped to clarify our contribution to the literature. |
| 2 | Reviewer #2: I want to thank the author(s) for this incredible undertaking and the exploration of multiracial identity formation (from a retrospective point of view) and their proposition of a framework to understand multiracial identity development. I appreciate that the author(s) took a qualitative approach with many interviews that were also geographically varied. | Thank you so much for your kind and supportive words about this manuscript. |
| 2 | The author(s) are examining multiracial identity development and its evolution over the life course, and so I would like to see more focus in the literature review that addresses how racialization of people of Color can often create psychological conflict and tension. That is, what the individual internally subscribes to as an identity and culture is not necessarily how the world will treat them because society ascribes an identity to them – essentially racializing them based on physical features and anything that can differentiate them from being white. Often this racialization against the individual is to the “lowest” racial group. | Thank you for raising this critical point. We added this into the introduction as we agree that this discordance between personal identification and how others identify is critical to understanding the identify development process and the impacts of that identity. The participants often discussed their struggles with this discordance. |
| 2 | Toward this idea, I think it would be important to talk about racial trauma and how that can be intergenerational (from parent/caregiver) to child and something that the child holds onto across their lifespan. I wonder if the author(s) asked about this. If not, why? | We agree that racial trauma is an important topic that should be further explored in this context. However, we did not feel comfortable adding it into this manuscript because we did not ask about racial trauma in the interview guide. We will absolutely keep this in mind for future research. |
| 2 | How was the interview questions developed? Did the author(s) develop the interview items from something else or run the questions by any multiracial individuals? | We added the fact that the interview guide was based on the conceptual framework as well as previous literature. We did not test the interview guide on multiracial individuals but we did adapt the interview guide as we learned from interviews along the way. |
| 2 | For the quotes, while extensive, they could be trimmed down and made more concise. I would like to see a bit more concision related to the quotes and a bit more on how to make sense of the quotes. That is, how might we interpret the quotes from the participants? | Thank you for this comment – we went through and tried to ensure that all excerpts were as concise as possible without losing any important context/meeting. |
| 2 | I would like to see more on the methodology, the thematic analyses. Citations? Perhaps a reference to a previous study using the same method. | A citation on thematic analysis was included in the methods section. |
